# Qualitative evaluation of the Autism Behavior Inventory: use of cognitive interviewing to establish validity of a caregiver report scale for autism spectrum disorder

**DOI:** 10.1186/s12955-020-01665-w

**Published:** 2021-01-20

**Authors:** Gahan Pandina, Seth Ness, Jeremiah Trudeau, Sonja Stringer, Naomi Knoble, William R. Lenderking, Abigail Bangerter

**Affiliations:** 1 Department of Neuroscience, Janssen Research & Development, LLC, Pennington, NJ 08534 USA; 2 Department of Neuroscience, Janssen Research & Development, LLC, Titusville, NJ USA; 3 Department of Patient Reported Outcomes, Janssen Global Services, Raritan, NJ USA; 4 Evidera, Pharmaceutical Product Development, LLC, Bethesda, MD USA

**Keywords:** Autism, Cognitive interview, Caregiver-reported outcomes

## Abstract

**Purpose:**

The Autism Behavior Inventory (ABI) is an observer-reported outcome scale measuring core and associated features of autism spectrum disorder (ASD). Extensive scale development (reported elsewhere) took place, in alignment with the Food and Drug Administration’s patient-reported outcome guidance, to address the need for instruments to measure change and severity of ASD symptoms.

**Methods:**

Cognitive interviewing was used to confirm understanding and content validity of the scale prior to its use in clinical trials. Respondents were caregivers of individuals with ASD (N = 50). Interviews used a hybrid of the “think-aloud” and verbal probing approach to assess ABI’s content validity and participant understanding of the instrument, including: item clarity and relevance; item interpretation; appropriateness of response scales; and clarity of instructions. Audio-recordings of the interviews were transcribed for qualitative data analysis. The scale was revised based on participant feedback and tested in a second round of interviews (round 1 N = 38, round 2 N = 12).

**Results:**

In total, 67/70 items reached ≥ 90% understandability across participants. Caregivers were able to select an appropriate response from the options available and reported finding the examples helpful. Based on participant feedback, instructions were simplified, 8 items were removed, and 10 items were reworded. The final revised 62-item scale was presented in round 2, where caregivers reported readily understanding the instructions, response options, and 61/62 items reached ≥ 90% understandability.

**Conclusions:**

Cognitive interviews with caregivers of a diverse sample of individuals with ASD confirm the content validity and relevance of the ABI to assess core and associated symptoms of ASD.

## Background

Autism spectrum disorder (ASD) is a heterogeneous neurodevelopmental disorder characterized by social communication deficits (e.g., social reciprocity, nonverbal communication) and restrictive behaviors (RBs) resulting in significant functional limitations [1].

Performance-based assessments (e.g., Autism Diagnostic Observation Schedule, Second Edition) [2] and clinical interviews (e.g., Autism Diagnostic Interview–Revised) [3] are considered diagnostic “gold standard” measures, and parent-report measures are often included in these assessments. However, few parent-reported instruments are available that measure core symptoms of ASD (i.e., social communication, restrictive behaviors) with brief recall periods appropriate for use in clinical trials [4–6]. In addition, instruments used for diagnostic purposes do not necessarily have sufficient sensitivity or specificity to detect responses to treatment [7, 8].

**Fig. 1:**
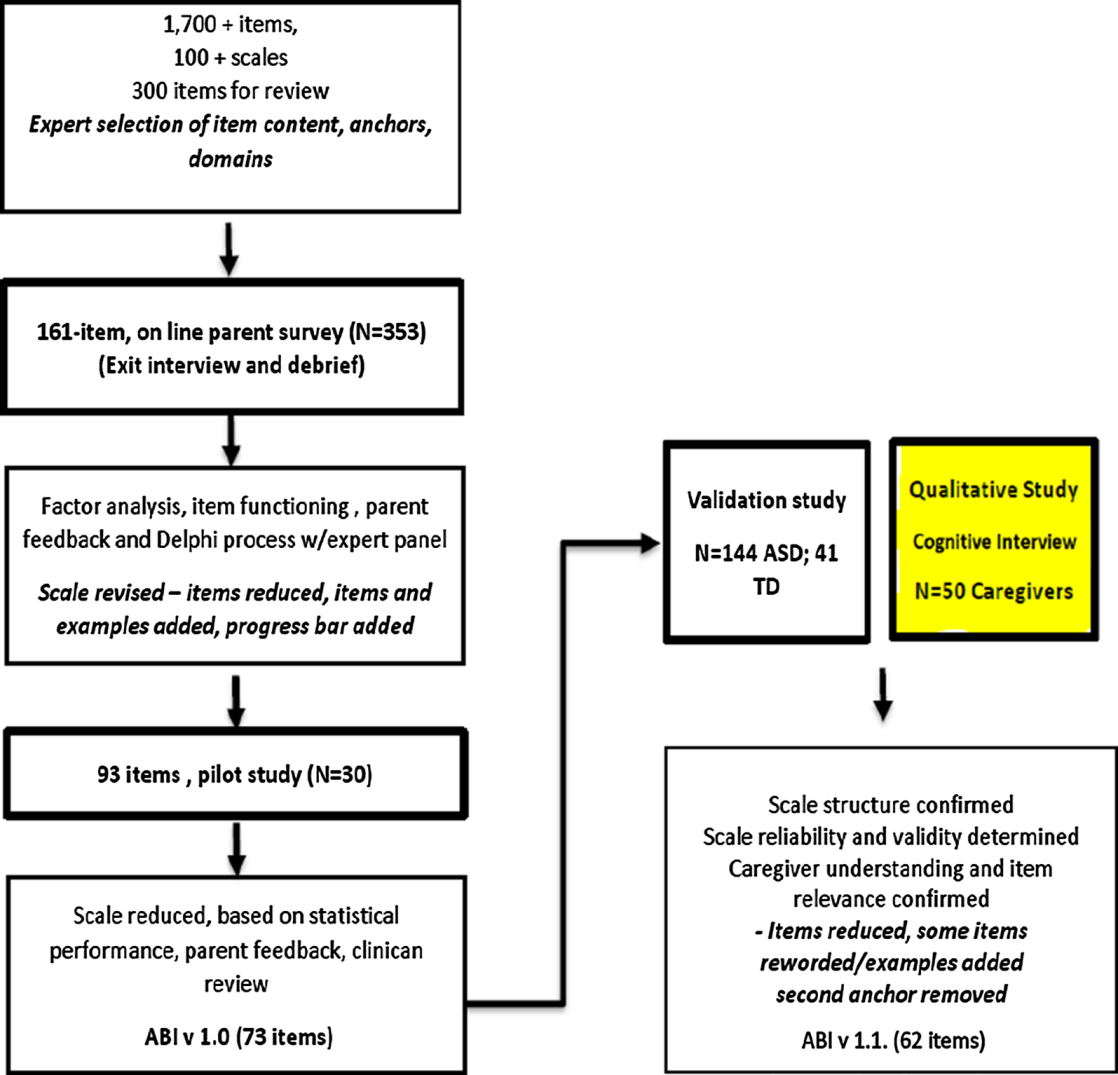
Development of the 62-item version of the ABI v1.1. *ABI* Autism Behavior Inventory, *ASD* autism spectrum disorder, *TD* typically developing

**Fig. 2:**
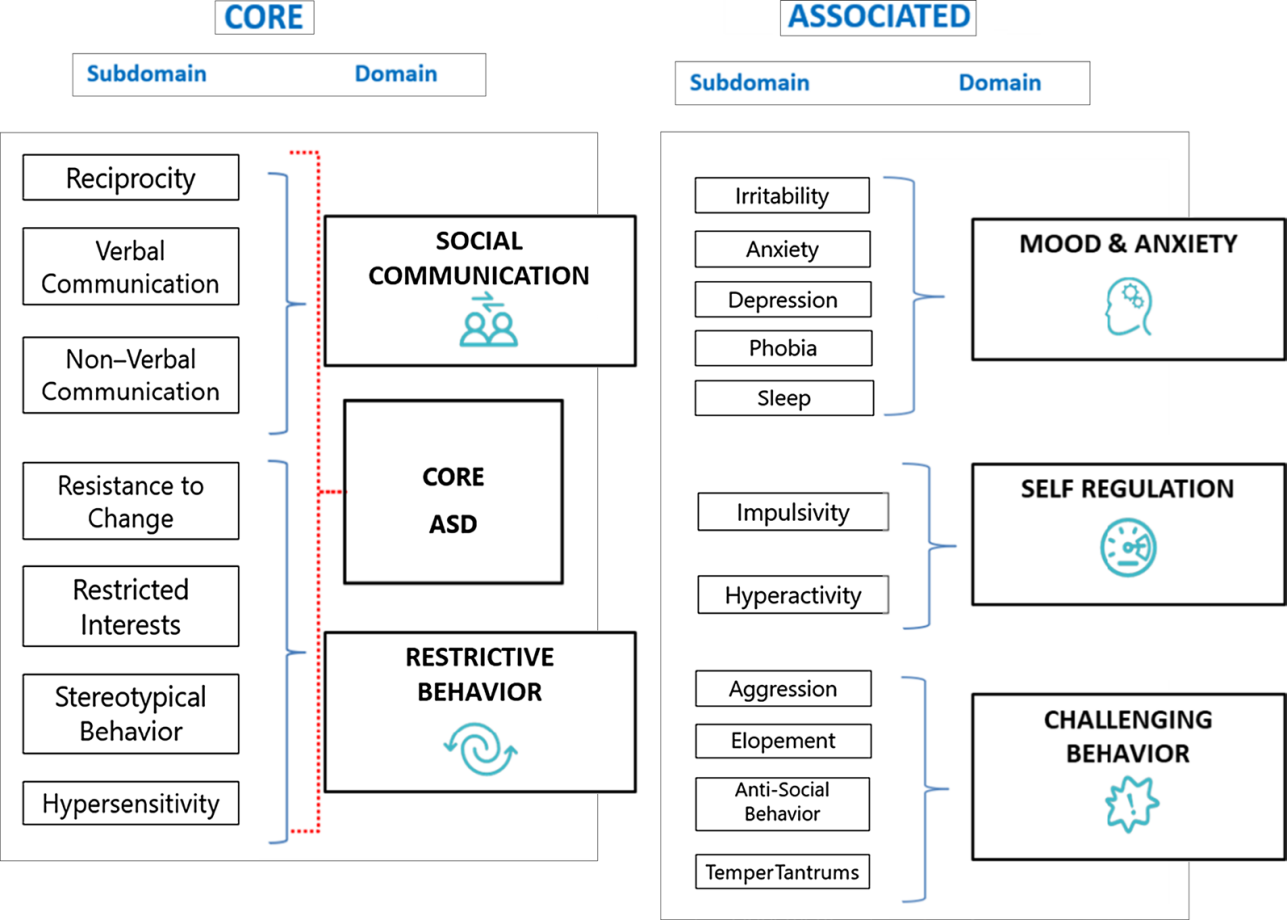
Overview of the ABI domains. *ABI* Autism Behavior Inventory, *ASD* autism spectrum disorder

The Autism Behavior Inventory (ABI) was developed in alignment with the Food and Drug Administration’s (FDA) Patient-Reported Outcome (PRO) Guidance [9] as a web-based rating scale for completion by caregivers to assess core and associated symptoms of ASD [10] (Fig. 1). The ABI was tested with a sample of 144 caregivers of individuals with ASD and demonstrated robust psychometric properties (NCT02668991) [11]. The ABI (v1.0) comprised 73 items across five domains (i.e., social communication [SC], RB, mood and anxiety, self-regulation, and challenging behavior) (Fig. 2). Regulatory review of the instrument led to some proposed changes, such as adaptation or removal of items to ensure suitability for all age groups and verbal abilities and reduction of two response dimensions to a single response. These changes were subject to further quantitative analysis to ensure the psychometric properties were maintained. Prior to the use of the instrument in a clinical trial it was important to ensure that respondents were able to understand and correctly interpret items, and that the instrument measured concepts relevant to the target group. Therefore, content validation was conducted using cognitive interviewing with parents and caregivers of individuals with ASD to confirm comprehension and acceptability of changes to the instrument and to ensure understanding and completeness of the concepts contained in the items [9].

Cognitive interviewing is a process whereby an interviewer employs a variety of techniques to prompt a participant to verbalize the thought processes that occur when interpreting an item and producing a response. For example, the participant may be encouraged from the start of an interview to “think-aloud” and spontaneously describe their thoughts as they respond to the questionnaire. An alternative approach is to use verbal probing, where specific questions are asked by the interviewer in order to elicit the thinking processes driving the response to the instrument [12, 13]. Use of probes can be helpful in cases where there is insufficient time for training in the “think-aloud” approach, and/or where participants find spontaneous description of their thought processes more challenging. Probes can be used throughout the task (Concurrent Probing), or can be used at the end of completion of the scale (Cognitive Debriefing) [14]. Procedural flexibility is viewed as one of the most useful features of Cognitive Interviewing [15].

Cognitive interviews are recommended for use in content validation of patient or observer reported outcomes [16, 17]. They can provide qualitative and quantitative analysis of whether participants understand question items, both consistently across participants and in the way intended by the researcher [18]. The approach also enables assessment of whether items and domains are relevant and important to the target population.

The objective of this study was to further develop evidence for the content validity of the ABI through cognitive interviews with parents and caregivers of a heterogeneous group of individuals with ASD.

## Methods

### Study design

This was a non-interventional, qualitative study consisting of interviews with parents and individuals who care for persons with ASD referred to as caregivers. The study design was structured-based on the recommendations of the International Society for Pharmacoeconomics and Outcomes Research Good Research Practices Task Force for establishing and reporting the content validity of PRO instruments to be used to support label claims [19], as well as the FDA PRO Guidance [9]. The FDA guidance indicates that evaluation of patient understanding through cognitive interviewing can contribute to evidence of content validity of items in the scale. A total of 50 participants across two rounds were recruited and interviewed from May 17, 2018 to July 11, 2018. Eligible participants were identified by an independent market research company via their proprietary databases, ASD advocacy support group network, and patient panels. Participants were required to be parents or caregivers of individuals with ASD aged 3 years or above, spends at least 3 h per day with the person with ASD, and read and understand English.

Caregivers were recruited and stratified based on age of the child as well as on the child’s verbal ability (minimal verbal functioning vs. higher verbal functioning). For the purposes of this study, minimal verbal functioning was defined as either no language, or use of signs, single words or two/three-word utterances. Higher verbal functioning was defined as the ability to form simple and/or full sentences. The aim was to achieve a balance across level of verbal functioning and age to adequately represent the broader population of individuals with ASD.

Institutional Review Board (IRB) approval of the study protocol and electronic informed consent were obtained from caregivers prior to completion of any study procedures. The study was approved by Quorum IRB.

### Cognitive interviews

For the purpose of these interviews a hybrid technique combining a “Think Aloud” approach with prompts as necessary were used to encourage verbalization of thoughts was utilized. A number of probe questions were asked, encouraging the caregiver to “think aloud”. Concurrent probing was used whereby the question was asked, followed by the caregiver answering the question and then the interviewer asking a probe question, if needed, and the caregiver responding.

**Table:** 

Examples of Probes Used:	
Can you repeat this question in your own words?	
What does this mean to you?	
How did you get to that response?	
What were you thinking about as you determined your answer?	
Can you say a little more about that?	
Was this hard or easy to answer? Can you tell me why?	
Was this difficult or easy to understand? Can you say a little more about that?	

Interviews were conducted via the web with the ABI presented on a shared screen using an online platform where caregiver participants and the interviewer could see and hear each other. Interviews lasted approximately 60–90 min and were audio recorded for transcription with study participants’ permission.

Trained interviewers carried out the interviews using the semi-structured interview guide. The interviews gathered participant feedback on the overall comprehension and clarity of the instructions, the individual items and examples, and item response options. Upon completion of the interview, participants were remunerated in the amount of $100 USD for their participation in the study.

Two rounds of interviews were conducted. The first round of interviews (round 1) was conducted with the 70-item version of the ABI, referred to as ABI_CI_v1.0. Round 1 was considered complete when saturation was achieved (i.e., interview responses were providing no new information about the items in the scale) [20, 21]. The study team, including professionals with clinical outcome assessment, clinical and ASD expertise, regularly reviewed and discussed caregiver feedback from interviews, and determined whether the saturation point had been reached. The review process also led to scale modifications that were implemented in the second interview round (round 2). A revised 62-item version of the ABI was utilized in interview round 2 and is referred to as ABI_CI_v1.1.

Not all participants were asked every question in the interview guide, in order to maintain interview flow and participant rapport, and to manage time constraints given the length of the semi-structured interview guide. Interviewers prioritized the core ABI domains of SC and RB, given their intended use as primary outcome measures in upcoming clinical trials. The three associated ABI domains were reviewed with caregivers as time permitted, and the presentation of these domains was counterbalanced across participants to ensure equivalent participant response coverage.

There is no fixed rule or field standard for the number of participants in cognitive interviewing for content validation of patient or caregiver reported outcomes. Some researchers indicate 12–15 participants per round is likely to be sufficient [22, 23], while others suggest a region or 20–30 participants to ensuring that saturation can be reached [21]. Given the number of items and possibility that not all participants would have time to cover all domains within the scheduled interview time, we planned for approximately 50 participants, anticipating that we could increase the number of participants for round 2 if required.

Similarly, there is no universal standard for participant comprehension, but with a clear, well-designed and simple instrument, a guideline of approximately 90% of respondents should be expected to understand the instructions, items, and response options [15]. We therefore adopted ≥ 90% participant-reported understandability as an indication of item understanding. This was used alongside further quantitative analysis of caregiver responses to determine exclusions or changes to items in the scale.

### Qualitative analysis

Audio files were transcribed, and a quality-assurance check was performed, primarily to remove all personal health information found within the transcripts, and to correct any obvious transcription errors.

A content-analysis approach was used to analyze the cleaned transcripts using a coding dictionary and ATLAS.ti version 7.5.18 qualitative data analysis software [24]. Participant quotes were grouped and summarized by thematic code, and coding outputs were generated based on each utilized code. Frequencies of participant responses were calculated.

## Results

### Participant demographic characteristics

Sociodemographic characteristics of the sample of 50 caregivers and 50 individuals with ASD are presented in Table 1. The mean age of individuals with ASD as reported by their caregivers was 12.4 years (range 2–40 years). Among individuals with ASD, there were more males (*n* = 33, 66%) than females ASD (*n* = 17, 34%). Caregivers had a mean age of 42.3 years (range 26–49 years). Most caregivers were female (*n* = 44, 88%), non-Hispanic (*n* = 46, 92%), White (*n* = 30, 60%), and married (*n* = 26, 52%). Regarding caregiver level of education, more than half of the caregivers reported some college, but no degree (*n* = 26, 52%), and nearly half of the caregiver sample reported being employed full-time (*n* = 24, 48%).

**Table 1:** Sociodemographic characteristics of caregivers of individuals with ASD

Characteristic	Total (*N* = 50)
Age of individual with ASD (years)	
Mean (SD)	12.4 (8.15)
Median [range]	12 [2–40]
Gender of individual with ASD, n (%)	
Female	17 (34)
Male	33 (66)
Age of caregiver (years)	
Mean (SD)	42.3 (8.88)
Median [range]	43 [26–59]
Gender of caregiver, n (%)	
Male	6 (12)
Female	44 (88)
Ethnicity of caregiver, n (%)	
Not Hispanic or Latino	46 (92)
Racial background of caregiver, n (%)	
White	30 (60)
Black or African American	14 (28)
Other	6 (12)
Education status of caregiver, n (%)	
Secondary school	1 (2)
Some college	26 (52)
College degree	15 (30)
Postgraduate degree	7 (14)
Doctorate degree	1 (2)
Employment status of caregiver, n (%)	
Part-time work	8 (16)
Full-time work	24 (48)
Homemaker	16 (32)
Retired	1 (2)
Disabled	1 (2)

*ASD* autism spectrum disorder, *SD* standard deviation

Clinical characteristics of the individuals with ASD, as reported by caregivers, are found in Table 2. Most individuals with ASD were diagnosed by a psychologist/neuropsychologist/psychiatrist (*n* = 23, 46%), or a pediatrician/primary care provider (*n* = 15, 30%), and the majority received their diagnosis between the ages of two and three years (*n* = 28, 56%). About half had fluent language (“speaks in full sentences,” *n* = 21, 42%), and the majority had at least one comorbid diagnosis (*n* = 32, 64%). Most individuals with ASD (*n* = 47, 94%) had had no significant changes in their condition in the past month, while three (6%) were reported to have had significant changes (two symptoms improved, one not known). For individuals with ASD still in school, the most commonly reported educational placements included regular classroom (*n* = 13, 26%) and a self-contained classroom (*n* = 9, 18%). Other than the higher proportion of females with ASD in our sample, these demographic and clinical characteristics are broadly similar to those of participants commonly reported and seen in clinical trials [25]. Saturation was reached after 38 caregiver interviews (in round 1) and 12 additional caregivers (in round 2) participated in interviews using the revised scale.

**Table 2:** Clinical characteristics of individuals with ASD

Characteristic	Total (*N* = 50)
Age at diagnosis, n (%)	
≤ 3 years	31 (62)
≤ 9 years	16 (32)
≤ 14 years	3 (6)
Level of language, n (%)	
No language	4 (8)
Puts signs or picture exchange together to make simple sentences	7 (14)
Single words/2- to 3-word utterances	10 (20)
Uses simple sentences	8 (16)
Speaks in full sentences	21 (42)
Other conditions, n (%)	
ADD/ADHD	12 (24)
Anxiety	3 (6)
Epilepsy/seizures	2 (4)
Global development delay	2 (4)
Intellectual disability/learning disability (e.g., math, reading)	8 (16)
Sickle cell	1 (2)
Speech disorder	1 (2)
None	18 (36)
Other: (e.g., behavior issues, insomnia)	3 (6)

*ASD* autism spectrum disorder, *ADD/ADHD* attention deficit disorder/attention deficit hyperactivity disorder

## ABI content validation

### Overall content validity

Analysis of the transcripts across items was carried out to determine consistency of responses between caregivers, and confirmation that the item conveyed the intended meaning. Qualitative analysis involved identifying the experiences, description, and perceptions that went into the respondents’ answers.

This analysis was used alongside qualitative analysis of whether respondents indicated an item was understood to determine whether wording changes, example additions, or item removal was appropriate.

The box below shows examples of the “think-aloud” responses for a specific item, “Has difficulty being flexible”, with the example “Has a hard time changing his/her mind”. Responses demonstrate differences in approach, with some participants (example 1 & 2) requiring minimum prompts to “think-aloud” and others (example 3 & 4) where the prompts were used to elicit responses **(interview questions are in bold font).**

**Table:** 

**Has Difficulty Being Flexible**	
Example 1& 2: Participant spontaneously thinks out loud.	
001–003: Has difficulty being flexible—example, has a hard time changing his/her mind—yes, that is “very often”.	
**That’s also (a response option of) “very often,” okay**	
001–003: Yeah, she is very in a routine. If anything is out of place, the smallest thing will cause a meltdown. Like she knows right now school is Monday through Friday, she knows that she gets up at the same time every day. Um, in terms of eating right now, that’s always been a little bit flexible, but other than that everything stays the same. So, like when—say, for instance, she didn’t understand summer vacation, when the kids are out of school. She was upset that she couldn’t go to school, because that’s become a routine to her.	
022–007: Has difficulty being flexible—has a hard time changing his or her mind. Uh, yeah, that would be “often”. That one’s really straightforward, too, you don’t need to change that at all. These kids are, you know, they just—things change and they have trouble moving with it—that’s the best way I could say it.	
**Example 3 & 4 Participant requires more prompts to think out loud**	
001–005: Has difficulty being flexible. Example, has a hard time changing his or her mind.	
**What would you select for your answer, and why?**	
001–005: Um, I would answer “never”.	
**Never. And why is that?**	
001–005: Um, my son is not really hard to deal with, like when we need to go out, uh, like we need to go out, it’s like right now, then even if he’s still watching TV, most of the time or most of the day he just watch TV, and when I tell him we need to go out, we need to go somewhere, even though he act like he don’t understand, but when I try, start changing his clothes, put his shoes on, he don’t really seem to get upset. He just, um, he just let me take him to wherever.	
002–010: Yes, I’m there. Has difficulty being flexible. Um, not really. Maybe once in awhile. I would say “sometimes” again.	
**Why would you say that? What’s your reason for that?**	
002–010: Because he, he knows the kind of society that we live in. He knows our day to day lives are constantly changing. What I find myself doing is saying to him, this weekend, I don’t know, we’re singing at the, we sing in church. We’re singing at the 8:00, we’re singing at the 9:30, we’re singing at the 11:00, we’re, you know, I will basically have to tell him what’s going on, or I will tell him when his father is working and where he will be, or if we’re able to go on vacation and where that might be. Um, and I feel as though the more I prepare him and communicate with him, the flexibility is better. So I will have to say to him, I know your violin lesson is on Friday at 4:30, but your teacher, [name removed], needs you to come today at 3:30, and he’ll say but it’s on Wednesday, and I’ll say yes, it’s Wednesday and we need to do it today, so let’s get ourselves together and get over there kind of a thing.	

### ABI Instructions

Participants were asked to comment on their impressions of the overall ABI instructions. Almost all participants that were asked to provide comments (*n* = 36/37, 97%) stated the instructions were “easy” and provided a good generalized overview of the questionnaire.

**Table:** 

**Are the instructions clear and easy to understand?**	
001–020 (Round 1): Um, I mean they’re pretty simple instructions. They don’t—they didn’t overuse words.	
002–012 (Round 1): Uh, pretty much, yeah—just wanting to know how—like how often something happens and, um, if you can’t do that, how the—the quality is, so, yeah, it’s pretty straight—straightforward, I think.	
002–003 (Round 2) …I think it’s a good way to start a study like this, so I think, as you said, there’s going to be some repetitive questions, so it gives you a good idea, it’s more of a generalized thing at the beginning and may be go into a little more detail as we move on.	

Some participants indicated more clarification was needed to clarify the two rating categories in the instructions. Six participants indicated that the term “dimension” could be changed to enhance understanding.

**Table:** 

001–015 (Round 1): Uh, not really, uh, I would—personally, I would take out the demen—dimensions and I would put, um, examples.	
001–008: (Round 1) Um, yeah. I—I mean, when I—I guess for the word ‘dimension,’ I was kind of thinking like what do they mean by ‘dimension’? Um, I’d say maybe like ‘factors’ or maybe a different word, um, to kind of describe what frequency and quality means.	
002–005 (Round 1): …Now I get it, there’s two different sets of questins, some will be asking you regarding frequency. Yeah, so maybe if they clarified that a little bit in the beginning it wouldn’t be so confusing, but these options are not confusing, these are easily understandable.	

As a result, the term “dimension” was removed from the instructions, and the description of the “quality” response was reworded.

### Recall period

Participants also provided feedback on the recall period of over the past 7 days. All participants who were asked to describe their understanding of the recall period (*n* = 42) were able to successfully explain it as intended, although there was some slight potential for confusion.

**Table:** 

**When you think of over the past week, what days do you envision?**	
001–005: From Monday to Sunday.	

In order to clarify the recall timeframe, after the first round of the ABI interviews, the wording was modified from “over the past week” to “over the past 7 days”.

**Table:** 

**What do you think over the past 7 days means?**	
002–006 (Round 2): Well, if today is Thursday, from last Thursday to this Thursday.	

### Response options

Participants were asked a series of follow-up questions to assess general understanding and conceptualization of the response options. For example, most participants (*n* = 38/41; 93%) stated the response options for both domains were easy and clear and most stated they did not have any suggested changes. For those who had difficulty with the response scale (*n* = 3/41; 7%), they indicated the option “with support” lacked clarity, the overall options were less clear than the frequency responses and had difficulty in differentiating “with support” vs. “with some reminders.”

**Table:** 

**All right. Are these response options clear and easy to understand?**	
002–006 (Round 1): Yes, they are. I think they are clear. They are pretty straightforward.	
001–028 (Round 1): Okay, um, let’s see, not at all means that it doesn’t even register to them. Um, with support would be somebody having to take them over to it or somebody physically showing them how to. With some reminders would be just a verbal prompt, like I would tell him, hey, [name removed], listen or [name removed], there’s so and so, without actually taking him over to the person. And then without help, they do it on their own.	
**What are your thoughts on these response options just over all?**	
001–028 (Round 1): Um, I like those—overall, they’re good.	
**So in your own words what does not at all mean to you?**	
001–009: Um, you never seen it, you don’t know nothing about it.	
**With support?**	
001–009: Uh, I guess with either prompts or the help of an adult or a little bit of guidance.	
**With some reminder?**	
001–009: Um, I guess verbally speech or possibly point at something to indicate what you’re trying to say.	
**Without help?**	
001–009: Um, totally independent, without any type of reminders or adult supervision.	
**And what do you think of these response options, from ‘not at all’ to ‘without help’?**	
001–006: They’re perfect. It explains it exactly. I mean, there’s an entire, um, umbrella within each category, but it definitely is the only categories you could have.	

### Overall response

In round 1 of interviews, 67/70 items reached ≥ 90% understandability across participants. Two items in the SC domain and 1 item in the RB domain were understood < 90% of the time. Table 3 shows responses for all items in these two core domains. For the associated domains all items reached ≥ 90% understandability.

**Table 3:** Percentage of caregivers’ understanding in core domains of SC and RB

Domain		Understanding	
Social communication		Round 1 n (%)	Round 2 n (%)
1	Responds to familiar things, *e.g., when a particular song is sung, when a familiar name is mentioned*	30/33 (85%)	Removed^a^
2	Shows appropriate affection towards familiar people	32/33 (97%)	12 (100%)
3	Shows an interest in what other people are doing	31/32 (97%)	11/12 (92%)
4	Responds to attempts to initiate social interaction	28/31 (90%)	12/12 (100%)
5	Gives things to others in order to get help, *e.g., brings you a box he/she can’t open*	28/30 (93%)	12/12 (100%)
6	Is flexible when playing with others or taking part in social activities	30/31 (97%)	10/10 (100%)
7	Is creative or imaginative in play or other activities, e.g., *make believe play or has new or original ideas*	31/31 (100%)	11/11 (100%)
8	Is able to take turns in conversation, *e.g., responds to and builds on what has been said, using speech or signs or gestures*	29/31 (94%)	11/11 (100%)
9	Directs facial expression towards other people to communicate feelings, *e.g., gives eye contact, shows emotion on face*	31/31 (100%)	10/10 (100%)
10	Offers information about his/her own thoughts or feelings, *e.g., able to talk or sign about what he/she is thinking and feeling*	26/26 (100%)	11/11 (100%)
11	Waves ‘hello’ and ‘goodbye’	27/27 (100%)	11/11 (100%)
12	Uses common gestures, *e.g., nods, shakes head*	27/28 (96%)	11/11 (100%)
13	Combines gestures with vocalizations to enhance communication, *e.g., uses actions and words to get point across*	25/25 (100%)	11/11 (100%)
14	Use tone of voice appropriately, *e.g., tone changes according to what he/she is saying*	25/26 (96%)	11/11 (100%)
15	Responds to other people’s emotions, *e.g., notices or comments on how others are feeling*	27/27 (100%)	10/11 (91%)
16	Looks when he/she is called or praised	27/27 (100%)	9/11 (82%)
17	Looks where another person is looking or pointing	27/27 (100%)	10/11 (91%)
18	Shows pleasure in shared interactions, *e.g., enjoys doing things with people*	26/27 (96%)	11/11 (100%)
19	Uses facial expressions that are appropriate to the situation, *e.g., looks sad when someone is hurt, smiles when happy*	26/26 (100%)	10/11 (91%)
20	Resists affection from familiar people	25/26 (96%)	11/11 (100%)
21	Shows inappropriate affection towards unfamiliar people, *e.g., hugging people that he or she does not know*	24/25 (96%)	11/12 (92%)
22	Has difficulty interacting with peers, *e.g., finds it hard to make and keep friends*	24/25 (96%)	11/12 (92%)
23	Says socially inappropriate things OR makes inappropriate social approaches, *e.g., will tell people they have a large nose, touches or strokes clothes or body parts*	26/27 (96%)	10/11 (91%)
24	Attends to parts of sentences and misinterprets whole, *e.g., focus on one or two words and misses the point*	14/22 (64%)	7/11 (64%)^a^

Restrictive behaviors			
25	Gets upset over small changes in routine	26/26 (100%)	12/12 (100%)
26	Has difficulty being flexible, *e.g., has a hard time changing his/her mind*	24/24 (100%)	12/12 (100%)
27	Resists trying out new things, *e.g., won’t go to new places, avoids new foods*	24/24 (100%)	12/12 (100%)
28	Insists on doing things the same way each time	23/23 (100%)	11/12 (92%)
29	Is fixated on certain topics or activities and unable to move on	24/24 (100%)	12/12 (100%)
30	Has an unusually narrow range of interests	21/24 (88%)	11/12 (92%)
31	Repeats /echoes what others say, *e.g., immediately repeats words or phrases*	23/23 (100%)	12/12 (100%)

*RB* restrictive behaviors, *SC* social communication. Please note more than 90% of caregivers understood all items in the associated domains (not shown)

^a^Items with < 90% understanding in round 1 that were reworded or removed for round 2 of interviews

Items with < 90% understanding were reworded or removed for round 2. In addition, if follow up comments from caregivers indicated confusion in response or similarity and overlap of items, these items were considered for removal or rewording.

### Use of examples

The ABI contains some items with examples, which were included based on quantitative and qualitative feedback in previous rounds of instrument development. Caregivers provided feedback regarding the examples either spontaneously, or in response to a prompt. The overall response to the inclusion and utility of examples was positive. It was also confirmed that some items were appropriate and easily understood without examples. Some caregivers suggested other items for which an example might be helpful. These suggestions were discussed by the scale development team and, where appropriate, examples were added to items for round 2.

**Table:** 

**What makes this question clear and easy to understand?**	
001–014 (Round 1): Well, that little example in there, helped it a lot.	
001–025 (Round 1): Um, I think the question is very clear and, um, the example, I think, is actually very good to back it up. I think the example needs to be there, and it’s a very good example.	
002–008 (Round 1): Yes. With, with the example. I think without the example, it would be difficult to understand.	
**Is the question itself overall clear and easy to understand?**	
001–006: It is. It’s clear and easy to understand, but I do think people would be like hmmm? So I like that. The—the example really just spells it out.	
001–025: Actually, I do—I love the examples, because like before it’s like parent-speak, it’s not clinical.	

### Item changes

Items changed as a result of the participant comments are shown in a tracking matrix (Table 4). Changes included rewording of items, where participants indicated wording was confusing (e.g. attends to parts of sentences, shown below) or where follow up comments from participants indicated some differences in understanding from expected meaning. Other changes included the addition of an example or removal of an item. Changes were then presented and confirmed in round 2.

**Table 4:** Modifications made to the ABI following round 1 of cognitive interviews

Wording presented in round 1	Final wording presented in round 2	Rationale
Social communication		
Shows an interest in what other people are doing	Pays attention to or notices what other people are doing	Re-worded to clarify that this is more about general awareness of people and less dependent on what people are actually doing^a^
Responds to attempts to initiate social interaction	Responds positively when others try to start social interactions	Re-worded to provide clarity^a^
Combines gestures with vocalizations to enhance communication, *e.g., uses actions and words to get point across*	Combines body language with words or sounds to support communication, *e.g., uses actions and words to get point across*	Re-worded to simplify so it was easier to understand^a^
Attends to parts of sentences and misinterprets whole, *e.g., focuses on one or two words and misses the point*	Attends to parts of sentences and misinterprets whole sentences, *e.g., focuses on one or two words and misses the point*	The term “sentences” was added after “whole” to capture the intended meaning of the item^a^
Resists affection from familiar people	Resists affection from familiar people *e.g. pulls away from family members/close friends or rejects verbal displays of affection*	Examples were added to capture the intended meaning of the item^a^
Responds to familiar things, *e.g., when a particular song is sung, when a familiar name is mentioned*	Removed	Removed at it was considered unsuitable for older individuals and some caregivers had difficulty interpreting^b^
Restrictive behavior		
Has mannerisms or odd ways of moving her/his hands or fingers, *e.g., flapping or moving fingers in front of eyes*	Has mannerisms or odd ways of moving her/his hands or fingers, *e.g., moving fingers in front of eyes*	The term “flapping” was removed from the example, this change was based on alleviating potential confusion of this item^a^
Makes repetitive movements, *e.g., flapping arms, rocking body, rolling head*	Makes repetitive movements, *e.g., flapping arms, rocking body, rolling head, spinning or tapping objects*	The example “spinning or tapping objects” was added to the example to provide clarity to better understand the item. It was combined with below item^a^
Attempts to harm him/herself	Behaves in a way that can cause injury to self, *e.g., biting self, picking skin, banging head*	Reworded and examples were added. The rationale to reword was based on providing simpler language to better understand the item clarifying this is not a suicide assessment item^a^
Has sensitivities to certain food textures	Is overly sensitive to certain food textures, *e.g., refuses food that is too crunchy, or too soft*	Reworded and an example was added based on providing clarification related to food allergies and/or sensitivities^a^
Has an unusually narrow range of interests	Has a very limited range of interests	“Unusually narrow” was removed and replaced with “a very limited” to provide clarity to the item as there was difficulty understanding the terms “unusually narrow”^a^
Uses objects repetitively	Removed	Removing this item was based on being potentially confusing. This was combined and covered in the repetitive movements item^b^
Mood and anxiety		
Is irritable and whiny, *e.g., grouchy, moaning when unhappy*	Is irritable, *e.g., grouchy, cranky, moaning when unhappy*	The term “whiny” was removed and an example was added due to the lack of understanding of the term “whiny”^a^
Worries about things, *e.g., generally worries about minor things, making mistakes, going to school*	Worries about minor things	The term “minor” was added and the example was removed for simplification^a^
Clings to adults or is too dependent on them	Clings to adults or is overly dependent on them	The term “too” was removed and replaced with “overly” based on providing clarification to better understand this item for younger children’s ordinary behavior of dependence^a^
Complains about physical problems without a known medical reason, *e.g., complains about aches and pains which may not be there*	Removed	Removed due to the potential for this item to capture typical child behavior rather than mood or anxiety concerns. Additionally, this item was removed due to ambiguity if the child is non-verbal^b^
Looks worried or concerned, e.g., has a fearful worried expression	Removed	Removed based on reducing the number of items in this domain and low frequency of occurrence (from this study and previous data)^c^
Gets upset when separated from a caregiver	Removed	Removed based on similarity to clings to adults or is too dependent on them^c^
Challenging behavior		
Runs away	Runs or wanders away, *e.g., does not stay in the place they should be*	The phrase “or wanders” and examples were added to provide clarity to the item so that it is interpreted as “wandering or running away”^a^
Is mean to animals, *e.g., pulls pet’s tail, shoves, kicks*	Removed	Removed based on the potential to capture behaviors observed during childhood rather than challenging behaviors associated with ASD. It was also removed because of the low incidence of the behavior, thus the potential to change in response to intervention^c^
Hits or kicks	Removed	Removed based on the potential to capture behaviors typical during childhood rather than challenging behaviors associated with ASD. This item was also considered to overlap with physical aggression^c^
Screams, yells, and cries	Removed	Removed based on the potential for this item to capture behaviors typical of children rather than challenging behaviors associated with ASD and similarity to other items^c^

*ABI* Autism Behavior Inventory, *ASD* Autism Spectrum Disorder

^a^Item reworded

^b^Item removed due to potential for misunderstanding

^c^Item understood but removed for other reasons (low frequency/limited applicability/overlap)

**Table:** 

**Attends to parts of sentences and misinterprets whole?**	
001–020: Not clear at all [laughter]. That one’s not clear at all. E.g., focus on one or two words and misses the point. Yes, I understand the example, however the question is pretty odd; attends to parts of the—of sentences and misinterprets whole. I—I feel like it’s not really a whole sentence or a question, it’s just—it’s not clear.	
001–003: Oh, misinterprets whole—um, focuses on one or two words and misses the point. Attends to the appropriate—no, attends to parts of the sentences and misinterprets the whole—misinterprets the whole what? The whole sentence or misinterprets...	
001–006: I don’t like the way it ends here. I don’t like the way it ends with the word ‘whole.’ I just think that’s very like—I get it, but I think—I don’t think everybody will.	

### Removal of items

In three cases, an item was removed because of participant difficulties with understanding. When considering removal, several factors were considered, including overlap with other items, comments from participants about suitability of an item for certain levels of verbal ability or age, number of responses at floor/ceiling, and perceived lower ability to detect change.

For example, “Uses objects repetitively” was removed because of the potential for differences in understanding by caregivers, and its similarity to other items in the domain. The item was combined with another item regarding repetitive and stereotypical behaviors in order to avoid confusion and reduce participant response burden (“Makes repetitive movements, *e.g., flapping arms, rocking body, rolling head, spinning or tapping objects*”).

**Table:** 

Uses objects repetitively	
**Is this one easy to understand or not?**	
001–024 (Round 1): Uh, when it says uses objects, I don’t know what objects, um, I don’t what objects that they’re talking about or—I don’t understand that one. But he is a repetitive person, he does things over and over again, so, um, I’m just thinking that, yeah, sometimes, it depends on what it is.	
002–003 (Round 1): Okay. Um, I guess here I would like to see some examples, because depending on the age of the child whether this is a toy or a blanket or stuffed animal, um, or it could be a tablet or a cell phone, um, I think it would just need to specify which, what type of object, or if it doesn’t matter that it’s any object.	

“Response to familiar things” was removed due to reported ambiguity by some caregivers. In addition, the high level of endorsement of this item by caregivers in this study along with previous data, may leave little room for change in response to intervention.

**Table:** 

Responds to familiar things	
**Is this question clear and easy to understand?**	
001–015: Not really. Uh, responds to familiar things, like I think it should be more clarified. Like what familiar things because a lot of people do a lot of different things. So, um, this question is not—um, it’s not clarified to me at all. I wouldn’t really know how to answer that question.	
001–014: Uh, responds to family [sic] things. When a particular song is sung, when a family member’s name is mentioned. I kind of don’t even understand the question myself.	

The item “Complains about physical problems without a known medical reason” was reported by three caregivers of younger and older minimally verbal individuals as not relevant or applicable to their child and was therefore removed.

**Table:** 

**Complains about physical problems without a known medical reason?**	
001–013: Okay. The—the only thing I’m going to say about this one is when you use the word complain, that to me says speech-wise, you know, verbal, verbally. He cannot do that, the only thing he can do is if something is bothering him, you know, if his finger hurts or whatever, he’ll come up and show it to you. But, um, so that’s the comment I would make on the question, um, or the, you know, actual item.	
001–009: Well, again, she’s not talking so, uh, uh, I don’t know.	

### Overall impressions

When asked about overall impressions of the ABI, participants indicated the questions were applicable, straightforward, and presented in language that was respectful of caregivers for individuals with ASD. Examples of responses included the following:

**Table:** 

**What are your overall impressions of the ABI?**	
001–003: The wording is pretty good, um, they are, like I said, self-explanatory. Um, you’ve given them to me in the most simplest [sic] terms that is the most easiest to understand, especially for a new parent or a parent that doesn’t understand, um, somebody that isn’t familiar with a child that has autism or who is on the spectrum.	
001–006: I mean, obviously, these are good questions.	
001–015: No, there was no questions, that’s—I thought that the time—these questions were very good in, um, helping parents like myself and others with, you know, autistic kids to, you know, better help, you know, understand the level of learning and training that they’re getting.	
021–002: Yeah, I guess my only comment, like I was just thinking about it with my son, if I was given something like this to kind of chart his behavior, I thought it was good.	
022–003: Someone who has a child helped write this, I think, which is great. **[Laughs]** Or a professor who had some very good knowledge, because the questions are really on point.	
001–024: “No, they were pretty good, yes, they were very good questions. It’s questions you don’t get on a daily basis, so it was very good—very good.”	
002–003: “… Uh, I think the questions are written very well, very clearly. …Uh, I don’t think you’d insult any parents with children on the spectrum, I think it was done very respectfully. I’m very interested…”	

## Discussion

This study was designed to confirm the content validity and applicability of items in the Autism Behavior Inventory (ABI) for caregivers of individuals with autism spectrum disorder (ASD), with differing language ability and ranging in age, from 3 years to adult. Participants represented a diverse range of education levels. Most participants were mothers, consistent with expectations for primary caregivers of individuals with ASD.

Response to the first version of the ABI was positive with > 90% of caregivers reporting understanding all but 4 of the items presented. Caregivers further demonstrated understanding of the ABI item content consistent with each other and with the expectations of subject-matter experts (clinicians and scale development professionals with experience in ASD) through the “think-aloud” approach. The instructions were reportedly clear, although some caregivers expressed a preference for a frequency rating to be used throughout, caregivers reported that the response options were appropriate, and they were able to provide responses to items using the 4-point scale.

Simplification of the response scale to a single type of response option (frequency) was discussed within the development team, but subject matter experts indicated that the quality scale assessed a different measure of social communication ability that was not captured by frequency count only. Caregivers were more familiar with a frequency response option but were able to use the quality response option and found it appropriate in most cases.

The ABI contains a proportion of items with examples. These examples were added during the development of the instrument in cases where qualitative or quantitative analysis had indicated potential for misunderstanding. The use of examples was found to be especially helpful by caregivers to interpret items and provide an appropriate response, for example, making the scale seem less ‘clinical’ and more parent-friendly.

Caregivers reported the items and the survey as a whole to be relevant and appropriate to the individual with ASD, covering the kinds of behaviors that they were living with day-to-day. This was established through analysis of examples that caregivers gave of the relevance of behaviors to their child, and also through feedback given, sometimes spontaneously and in the opportunity for comments at the end of the interview. There were no suggestions of addition of items, and items such as sleep and food sensitivity which had been added in response to previous caregiver suggestions were validated by this group as being important items. Items in core and associated domains identified as valid in the ABI are also consistent with items and areas of importance identified in other qualitative studies of caregivers with ASD [8, 26].

## Limitations

As indicated, the sample over-represented females with ASD relative to the gender distribution within the general population of those with ASD (2:1 male: female in our sample vs. 5:1), and this may have impacted the interpretation of the items. Similarly, despite counterbalancing efforts, the sample was not robustly representative of minority populations. The study participants viewed an online pdf of the ABI rather than the actual web-based form itself, which may have impacted participant responses and did not provide electronic usability evaluation, though information on the usability and acceptability of the online version of the ABI has been reported elsewhere [27, 28]. Finally, not all participants completed all items. However, it was ensured that a sufficient number of caregivers did complete items in each domain to be confident in the results, and a second round of interviews with the reduced scale enabled completion of more items by caregivers increased the robustness of findings. The lowest number of participants completing an item in the associated domains was 14 in round 1, and 7 in round 2. Therefore, each item from the associated domains had been reviewed at least 21 times in the course of the interviews. The consensus among the review team was that saturation had been reached for these items.

## Summary and conclusions

In summary, the hybrid cognitive interview process, using spontaneous “think-aloud” and prompts was successful in eliciting responses and feedback on the ABI. Analysis of the responses resulted in a revised 62-item instrument assessing five domains of functioning that demonstrated content validity with caregivers of individuals with ASD. The results of the cognitive interviews demonstrate that the ABI instructions, items, item examples, and response options comprise a content valid caregiver-reported instrument aligned with instrument development methods described in FDA’s PRO guidance [9]. In response to caregiver feedback, minor adjustments were made to the ABI, specifically the simplification of the instructions, removal of some items, simplification of the phrasing of some items, and inclusion of some behavioral examples. This instrument can be considered content valid across a wide range of verbal ability for children and adults with ASD, and for caregivers of individuals with ASD. Additional psychometric evaluation data will support the ongoing development and validation of the ABI for use in clinical trials.

## Data Availability

The datasets used and/or analyzed during the current study are available from the corresponding author on reasonable request. The Autism Behavior Inventory (ABI) v1.1 is available without charge for academic, research, and professional use, subject to terms and conditions. It can be downloaded in the USA from https://www.janssenmd.com/ (in the tools/psychiatry section) and accessed outside the USA via email request to autismbehaviorinventory@its.jnj.com.

## Abbreviations

ABI: Autism Behavior Inventory
ADD/ADHD: Attention deficit disorder/attention deficit hyperactivity disorder
ASD: Autism spectrum disorder
FDA: Food and Drug Administration
IRB: Institutional Review Board
PRO: Patient-Reported Outcome
RB: Repetitive behavior
SC: Social communication
SD: Standard deviation
TD: Typically developing
